# Haus der Barmherzigkeit: Birthplace of Geriatrics

**DOI:** 10.1007/s10354-025-01112-9

**Published:** 2025-10-13

**Authors:** Christoph Gisinger

**Affiliations:** 1 Medical University of Vienna, Teaching Hospital Haus der Barmherzigkeit, Seeböckgasse 30 A, 1160 Vienna, Austria; 2 Academy of Ageing Research at the Haus der Barmherzigkeit, Seeboekgasse 30 A, 1160 Vienna, Austria

**Keywords:** Nascher, Geriatrics, Gerontology, Aging, Ageing, 150th anniversary, Haus der Barmherzigkeit, House of Mercy, History, Medical specialities

## Abstract

Medical care for older adults has existed since the early days of medicine, but it was Ignatz L. Nascher’s coining of the term “Geriatrics” in analogy to pediatrics in 1909, inspired by his visits to facilities in Vienna and its tradition in establishing new fields of medicine, that gave rise to a new awareness. These facilities included the Lainz care facility, which had only been founded 5 years earlier and was modeled on the Haus der Barmherzigkeit founded in 1875, and included medical care and special design of the living environment. In a later article on the history of geriatrics, John E. Morley wrote in 2004, “Austria was a powerhouse … of geriatric care” and “the Austrian system … inspired Nascher to coin the term geriatrics.” An important foundation of this “system” was the Haus der Barmherzigkeit, which had been founded 30 years earlier and can therefore be regarded as the birthplace of geriatrics. After an eventful 150 years, the Haus der Barmherzigkeit is now ready to make important contributions to the more stable establishment and further development of the specialist area of geriatrics, with an excellent quality of care as well as research and teaching and concepts to make it more attractive to employees.

In many respects, Vienna is regarded as the birthplace of ideas and a source of inspiration, as the historian and senior editor of *The Economist* magazine Richard Cockett has illustrated in his book on many examples of modern life that can be traced back to Viennese impulses from the late 19th and early 20th century: “*Vienna—How the City of Ideas Created the Modern World*” [1]. Apart from chapters on art, architecture, philosophy, economics, social science, and others, medicine is only touched on in passing, particularly in the area of psychoanalysis.

## Vienna Medical School

In Vienna at the end of the 19th century, Theodor Billroth developed new surgical methods, and Johann von Mikulicz, Josef von Skoda, Otto Kahler, and Carl Wilhelm Hermann Nothnagel revolutionized internal medicine. The Vienna Medical School became a place of pilgrimage for physicians from all over the world, including around 200 medical doctors from the USA alone every year. The first eye clinic was established at the beginning of the 19th century, followed by a series of other specialized clinics. Ernst von Feuchtersleben became a pioneer of psychosomatic medicine and medical psychology with his *Textbook of Medical Psychology* published in 1845. Sigmund Freud was able to build on this and other outstanding Viennese neurologists and psychiatrists when he set out to revolutionize psychiatry with his reports on “hysteria” published from 1892 onwards. On January 17, 1896, Sigmund Exner, who was in close contact with Conrad Röntgen, demonstrated the first roentgenogram to the Society of Physicians in Vienna, a procedure that was to develop further in Vienna for individual clinical issues with astonishing speed [2].

## Morbus Viennensis

However, these “triumphs” at the cradle of modern scientific medicine cannot conceal the fact that in many cases, there could be no question of a “cure.” In 1867, one in four Viennese died of tuberculosis (also known as “Morbus Viennensis”). At that time, Vienna was a booming city and grew from 440,000 inhabitants in 1840 to around 900,000 in the 1870s and to 2.1 million in 1910, making it the third largest city in Europe after London and Paris. Founded in 1881—shortly after the Ringtheater fire—the Vienna Ambulance Service recorded a large number of calls for patients who had collapsed in the street with consumption, malnutrition, or exhaustion. Tuberculous bone and joint disease, rickets, and the consequences of accidents and infections led to a vast number of “cripples” who—just like mentally and physically disabled children and young people or older adults—were unable to work for a living or to provide for themselves [2].

## Haus der Barmherzigkeit

**Fig. 1:**
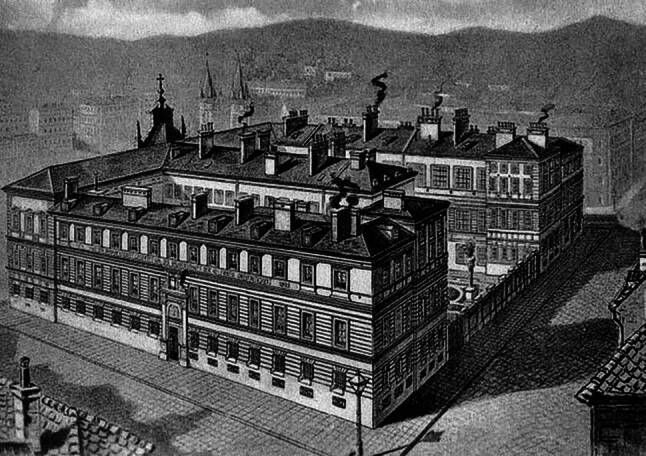
The Haus der Barmherzigkeit in Vienna, Währing, at the end of the 19th century

Those who could fall back on financial resources and, above all, family support, were lucky. Monasteries and public hospitals were soon overwhelmed by the growing number of poor people, not to mention medical care. In 1864, the Bruderschaft zur allerheiligsten Dreifaltigkeit zur Pflege armer Unheilbarer (Brotherhood of the Most Holy Trinity for the Care of the Poor and Incurable) was revived around the printing press owner Franz Eipeldauer, renewing an old pilgrimage tradition dating back to the plague era. Eventually, they were able to attract enough donors and sponsors to build the Haus der Barmherzigkeit (House of Mercy) in Vienna’s Währing district in 1875, which had to be expanded from an initial 22 beds to around 700 beds within only few years ([3]; Fig. 1).

## Providing medical care

What was remarkable and unique at the time was the fact that medical care was also provided from the outset. The majority of the people to be treated were adults—“incurable” increasingly meant “old” and “chronically ill”—and the physicians were able to gather and pass on their experiences of their complex clinical pictures. This impressed Karl Lueger (major of Vienna 1897–1910), controversial for his demagogic populism and antisemitism but formative for the city’s development, including the construction of the Versorgung Lainz (Lainz Care Home) in 1904, where medical care was also provided based on the model of the Haus der Barmherzigkeit.

## Ignatz Leo Nascher

When the Vienna-born New York physician Ignatz Leo Nascher visited his hometown in 1909, he also got to know Lainz and, at least indirectly, the medical care concept of the Haus der Barmherzigkeit. Under the impression of these experiences and in analogy with pediatrics, which had been established in Vienna since 1937 with the founding of the first children’s clinic, Nascher coined the term “geriatrics” for the first time in an article in the *New York Medical Journal* [4] immediately after visiting his native city. It says about an old person or patient that “Having an individuality of its own as clearly defined as childhood, with anatomical features, physiological functions, diseases, and their treatment differing from maturity it should be considered apart and distinct from maturity, and as a special branch of medicine. To such specialty I would apply the term geriatrics.”

## Austria: powerhouse of the emerging field of geriatric care

**Fig. 2:**
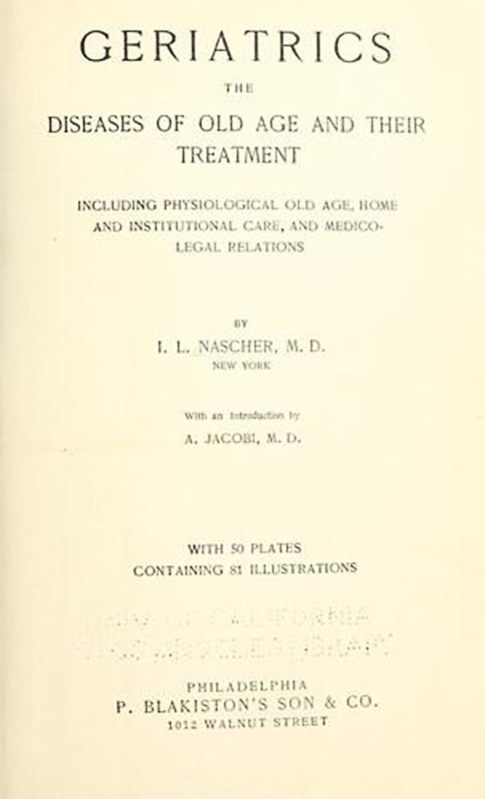
Title page of Nascher’s textbook Geriatrics—The Diseases of Old Age and Their Treatment, Philadelphia 1914 P. Blakiston’s Son & Co

In an article about the history of geriatrics, John E. Morley points out, “Austria was a powerhouse of the emerging field of geriatric care. It was the Austrian system that inspired Nascher to coin the term geriatrics.” [5]. In Nascher’s understanding of the term geriatrics, the physiological, pathological, diagnostic, therapeutic, and psychosocial aspects are emphasized, in accordance with the “Austrian system” mentioned by Morley, which was primarily influenced by the Viennese development and can be traced back to the medical care model of the Haus der Barmherzigkeit established in 1875 while also shaping the living environment. This is also expressed in a passage from Nascher’s textbook published in 1914 ([6]; Fig. 2), “at Lainz near Vienna, which the author visited, the inmates receive counters representing money which can be exchanged at a canteen on the grounds for beer or tobacco. To prevent the ennui which leads to melancholia, the inmates follow their vocation in the institution, as far as they are able, and go twice daily to the canteen which is fitted up as ‘bier stube’. They have a band, and in other ways their interest in life is maintained. They are naturally under restrictions, but they are at liberty to go and come at will within certain hours, and the depressing idea that they are paupers is not forced upon them” (textbook page 487).

## Haus der Barmherzigkeit overcomes crises

After the collapse of the Austrian monarchy in 1918, economic crisis in the 1920s/1930s, annexation of Austria by Nazi Germany in 1938, another World War, expropriation in 1939, and restitution only in the 1950s, the Haus der Barmherzigkeit was busy surviving and rebuilding the facility marked by bomb hits. Of course, the entire medical and social sector suffered in a similar way. So, it took until 1955 for the Österreichische Gesellschaft für Geriatrie und Gerontologie—ÖGGG (Austrian Society of Geriatrics and Gerontology) to be founded on the initiative of Walter Doberauer, who also published a textbook on geriatrics in 1961 [7]. By this time, the Haus der Barmherzigkeit had regained its reputation as a pioneer with the opening of a ward for patients with advanced multiple sclerosis and a focus on the care of patients with vegetative state (appalic syndrome). This reputation was also underlined by the fact that Pope John Paul II also visited Haus der Barmherzigkeit during his state visit to Austria in 1982. In the 1980s, thanks to the persistent efforts of the then Medical Director Franz Böhmer, the costs of medication were finally covered by social health insurance, and the diagnostic in-house facilities were expanded (endoscopy, X‑ray, ultrasound, dental treatment). These activities, experiences, and the model of Viennese geriatric care were also reflected in later textbooks by Böhmer [8, 9] and also Karl Heinz Tragl [10]. At the turn of the millennium, the structural infrastructure that had been hastily and sparingly repaired in the post-war period was no longer adequate in terms of hygiene, fire protection, and privacy. Therefore, the Haus der Barmherzigkeit decided to fundamentally modernize and renovate the entire building stock thanks to the support of the ecclesiastical protector Archbishop Christoph Cardinal Schönborn and under the chairmanship of the Institute Council Reinhard Krepler, who was also director of the AKH (Vienna General Hospital) at the time, in partnership with the city and federal state of Vienna and the federal state of Lower Austria which are responsible for financing operations in the form of a daily rate, taking into account the income recourse of the persons receiving care.

## Haus der Barmherzigkeit sets standards again

Since then, all facilities have been newly built and include two geriatric hospitals, five nursing homes, and 24 new facilities for people with severe multiple disabilities, comprising care of a total of around 1700 people and around 1650 employees from the various professional groups (Table 1). The geriatric hospital type is a hybrid form of hospital and long-term care facility with medical equipment comparable to that of conservative departments of acute hospitals and round-the-clock medical service, subject to the Hospital Act in terms of regulation and like a long-term care facility in terms of financing and length of stay (Tables 2 and 3), with the consequence that income recourse is possible for the persons cared for. The facilities operated directly by the City of Vienna itself were also built and operated according to these concepts a few years later.

**Table 1:** Haus der Barmherzigkeit’s facilities

Facility	Location	Beds/places	Staff	Remarks	Going into operation
*Geriatric hospitals*	In total	634	712	–	–
HB SBG	16th Distr. Vienna	364	416	Dept of Internal Med and Geriatrics and Dept of Neurogeriatrics	2005
HB TOK	22nd Distr. Vienna	270	296	Dept of Internal Med and Geriatrics and Dept of Gerontopsychiatry	2006
*Nursing homes*	In total	616	574	–	–
HB Am Maurer Berg St.-Josef	23rd Distr. Vienna	65	58	Extension to 130 beds by 2028 in implementation	2018
HB Clementinum	Kirchstetten, LA	126	131	Extension to 162 beds by 2028 in implementation	2003
HB Stephansheim	Horn, LA	143	140	Including hospice; additionally assisted senior living with 40 apartments in implementation by 2027	2014
HB Urbanusheim	Poysdorf, LA	120	116	–	2011
HB Stadtheim	Wr. Neustadt, LA	162	129	–	2017
*Facilities for people with severe multiple disabilities*	In total	443	461	–	–
HABIT assisted living communities	15 locations in Vienna and LA	161	255	Including two for children	From 2000 to 2022
HABIT Garconniere association	2 locations in Vienna	24	38	Another one in preparation	
HABIT day care	5 locations in Vienna and LA	191	131	–	
HABIT mobile care	Vienna	67	37	–	
*HEIG seminar center and administration*	16th Distr. Vienna	–	90	Academy for Ageing Research, communication, information technology, logistics, facility management, administrative back office	2019
*Total*	*All facilities*	*1693*	*1837*	–	–

*HB* Haus der Barmherzigkeit, *HABIT* Haus der Barmherzigkeit Inclusion Team, *HEIG* Heigerleinstrasse, *SBG* Seeböckgasse, *TOK* Tokiostrasse, *LA* Lower Austria

**Table 2:** Characteristics of Haus der Barmherzigkeit geriatric hospitals

Legal form	Hospital for the chronically ill in accordance with Section 2 and 3, Paragraph 1, of the Viennese Hospital Act
Financing	Daily rate fed from the federal care allowance, social assistance funds from the federal states, and income recourse of the person receiving care
Management	Collegial management consisting of medical director, director of nursing services, administrative director, and technical director
Medical service/specification	Round the clock: general medicine; daily in-house available internal medicine, neurology, psychiatry; weekly scheduled, almost all specialists available in-house as consultants on a weekly basis according to planning, including dental treatment (appropriate equipment and assistance available)
Healthcare professions	Certified nursing, physiotherapy, occupational therapy, psychology, psychotherapy, logopedics, social work
Equipment	Medical procedure room, X‑ray, ultrasound, endoscopy, slit lamp, dental treatment chair and accessories, etc.

**Table 3:** Geriatric hospitals, staffing characteristics

HB geriatric hospitals staffing	HB SBG	HB TOK
Employed physicians	16	12
Consultant physicians	11	11
Rehabilitative therapeutic specialists	20	16
Psychologists and psychotherapists	4	3
Certified nursing	122	62
Other nursing	223	180
Other personnel	20	12
*Total*	*416*	*296*

*HB* Haus der Barmherzigkeit, *SBG* Seeböckgasse, *TOK* Tokiostrasse

## Quality assurance and innovation

Of course, building is not the end of the story. Therefore, Haus der Barmherzigkeit placed much emphasis on quality assurance and the development of appropriate methods [11], and in 2004, ISO certification was achieved as the first institution of its kind. The next step was the introduction of comprehensive electronic care and health documentation in 2008, with the aim of improving the quality of care, supporting documentation, and learning from long-term data. Among other things, this made it possible to develop and continuously improve a method for better assessing the need for medical care in the form of a score, which was named the “Nascher-Score” in honor of Ignatz Leo Nascher [12]. In the area of the two geriatric hospitals, new concepts were made possible with the establishment of a separate department for neurogeriatrics in the Haus der Barmherzigkeit Seeböckgasse and a department for psychogeriatrics in the Haus der Barmherzigkeit Tokiostrasse, and new concepts for geriatric rehabilitation were developed. The conversion of an office wing in the Haus der Barmherzigkeit Seeböckgasse into a very special facility for very seriously chronically ill children with a limited life expectancy, the Fridolina children’s care “domicile” closed the circle in the relationship between pediatrics and geriatrics, which Nascher referred to and which is why he was able to win over Abraham Jacobi, the pioneer of pediatrics in the USA, for a foreword in his textbook. Another key focus was on human resources (HR), with creation of the strategic HR development staff position in 2018, which helped the Haus der Barmherzigkeit to come through the pandemic comparatively successfully and be subsequently repeatedly recognized as the most attractive employer in the Austrian health and social care sector by the business magazine* Trend* [13].

## Research in Geriatrics and Haus der Barmherzigkeit

Much of the research in the field of geriatrics in Austria comes from the organ disease-related established disciplines of medicine and hardly covers everyday geriatric life with the complexity of multimorbidity, interprofessional challenges, the possible use of assistance technology including robotics, and psychosocial and ethical issues. Unfortunately, Austria, as the former “powerhouse of geriatric care,” is structurally weak. Only in 2006 was the first and still structurally best equipped chair for geriatrics created by the private Paracelsus Medical University in Salzburg and filled by the neurologist Bernhard Iglseder. This was followed in 2011 by the Medical University Graz with the appointment of Regina Roller-Wirnsberger and in 2012 by the Medical University Vienna with Marcus Köller, although the latter professorship expired after just 5 years. In recognition of his activities and the potential of the research group set up at Haus der Barmherzigkeit with the Akademie für Altersforschung (AAF; Academy for Ageing Research), the author of these lines was appointed (parttime) professor of geriatrics at the Donau Universität Krems (University of Continuing Education Krems) in 2008 (now emeritus from this position at the end of 2021) and, as director of the institute, continues to ensure the further development of research and teaching with a very committed team. Research activities at the Haus der Barmherzigkeit include prevention and public health [14–16], clinical research [17–21], assistive technology [22–25], quality of life research, and related fields [26, 27]. More information can be found on the homepage of the Academy of Ageing Research [28].

## Teaching in Geriatrics and Haus der Barmherzigkeit

In the area of teaching, an international postgraduate four-semester master’s program (degree with the title MSc in Geriatrics, block courses) was developed in cooperation with the University of Continuing Education Krems (Danube University Krems) between 2007 and 2021, which over 100 physicians have completed over the years, as well as other certified programs on topics such as geriatric assessment, geriatric psychiatry, law and geriatrics, and others. At the same time, since 2008, there has been ever closer cooperation with the Medical University of Vienna in the area of a compulsory course for all first semester medical students (780 every year), with preparatory seminars in small groups, practical training, and supervisions all held in the Haus der Barmherzigkeit facilities. Since then, well over 10,000 (future) doctors have had this experience, which provides them with an opportunity for self-awareness in a medical geriatric setting to develop communication and social skills with patients and interprofessional interaction with corresponding accompanying research [29]. Other activities include the organization of a lecture series on geriatrics and many smaller training courses (for more, see altersforschung.ac.at/en). It is to be hoped that it will be possible to anchor geriatrics more firmly as a subject at medical universities, to include it more in the curriculum, and to strengthen it structurally, for example through professorships and cooperation with relevant institutions.

## Conclusion and outlook

Nascher coined the term geriatrics in 1909 after visiting his native city, inspired by the “Austrian system” and the then recently built large care home Lainz, which was very strongly influenced by the existing Haus der Barmherzigkeit founded in 1875. Therefore, it sees itself as the birthplace, or at least as the breeding ground, of geriatrics and feels obliged to contribute to the further development and firm establishment of geriatrics in Vienna and Austria—through exemplary quality of care, teaching, and research as well as the design of working conditions for staff to attract the best specialists to this important field. In view of the important role that Vienna has played in the development of medical and other fields, it is high time that geriatrics finally regains the importance it deserves in Austria. In his book, Cockett describes how Vienna created the modern world. The “modern” world of tomorrow is a world with an increasing number of geriatric patients, to which any of us can belong if we live long enough. As a society, we must prepare ourselves. The Haus der Barmherzigkeit is ready.
